# Greyhound morbidity and mortality in Australia: A descriptive analysis of reported data from regulatory racing agencies

**DOI:** 10.3389/fvets.2022.925948

**Published:** 2022-09-23

**Authors:** Vysie Chang, Kris Descovich, Joerg Henning, Rachel Allavena

**Affiliations:** School of Veterinary Science, University of Queensland, Gatton, QLD, Australia

**Keywords:** greyhound dogs, animal welfare, regulations and policy, racetracks, animal euthanasia, injury, fatality

## Abstract

Commercial greyhound racing is legal in Australia but controversial due to concerns around animal welfare. To make evidence-based recommendations of animal welfare standards, a comprehensive analysis of available data on race events, animal health, injuries and fatalities is required. We undertook a review of publicly available data and reports published by official greyhound racing bodies for the purpose of determining how morbidity and mortality events associated with dog training and racing could be benchmarked. 6 years of available data from stewards' reports, quarterly and annual reports were descriptively analyzed from New South Wales, Victoria and Queensland. Whole-of-life tracking for individual dogs was sparse. Although stewards' reports were published in all three states, the availability of aggregated quarterly and annual reports varied. When available these provided additional information such as injury incidents standardized per thousand starts. In Queensland, quarterly and annual reports provided an overview of greyhound mortality and morbidity rates. In contrast with Victoria, quarterly reports were unavailable and only annual reports were published, meaning quarterly trends could not be determined. Therefore, injuries categorized by severity that were routinely included in quarterly reports in Queensland and New South Wales were unavailable in Victoria. Our findings demonstrate that data recording and reporting practices must be standardized to accurately evaluate whether animal welfare standards are being met in the Australian greyhound racing industry. Our recommendation is to have national standardized reporting of injuries and deaths, and a publicly available database for whole-of-life tracking for individual racing greyhounds.

## Introduction

In the Australian context, greyhound racing is an established industry that has become a strong economic force firmly backed by prominent stakeholders. Although participation in any sport has its risks and benefits, animal racing is unique in the type of risks incurred by participating animals. Adverse consequences to the racing animal range from minor to catastrophic injuries, including death. Additionally, training practices can have significant welfare consequences. In 2015, an exposé broadcast by Australian media featuring video footage of inhumane and illegal greyhound training methods known as “live-baiting” generated heated public and political debate (1). Although historically the industry has had to manage ongoing criticism from animal protectionists, this media campaign highlighting animal welfare issues reached mainstream society. Subsequently, the greyhound industry received unprecedented scrutiny from the general public. As the racing industry relies on the use of animals as commodities for the pleasure of gambling patrons and potential financial gain of owners, the concept of social license plays a crucial part in any animal racing operation, and thus issues of public and political perceptions cannot be ignored (2).

Greyhound racing in Australia is regulated on a state level. The lack of a national regulatory body means that the control and regulation of greyhound matters varies state-by-state. Soon after the 2015 exposé was aired, official inquiries into the greyhound racing industry were conducted in multiple states. In 2015, QLD published the MacSporran Report, which was followed by NSW's McHugh Report in 2016 (3, 4). Recommendations put forth by MacSporran included the formation of an independent Statutory Body to ensure industry integrity, and the tracking of greyhounds from birth to leaving the racing industry. This would encompass tracking details of injuries and deaths that occur during the course of racing (3). In response to the 15 recommendations outlined in the MacSporran report, the QLD Government accepted them all and vowed to implement actions to address them (5). The State Premier of NSW took a stronger stance and made an unsuccessful attempt at banning greyhound racing in NSW altogether (6). Despite strong opposition against greyhound racing, it became clear how influential greyhound industry stakeholders were, and the ban was overturned. Even with the failed attempt at banning greyhound racing, NSW retained its intentions to improve greyhound welfare, industry oversight and transparency. Nevertheless, for an industry to simultaneously regain good standing with the community, maintain the trust of its patrons and quell unease of the stakeholders, at minimum, the baseline expectations of all parties must be met. Since banning the sport has proven complicated and unlikely, compromises must be made by the industry to ensure animal welfare standards are adhered to, allowing support of social license. The Greyhound Industry Reform Panel was established by the NSW Government on 11 October 2016 to address several of the concerns raised in the McHugh report (7). Recommendations outlined by the panel followed a holistic approach to ensuring animal welfare standards are met. These include suggestions for more stringent and enforceable rules, regulations, code of practice, to introduce new offenses and stronger penalties against animal cruelty, and to implement whole-of-life tracking of greyhounds (7). To decrease the number of unnecessary euthanasia, several recommendations have been given. This includes new requirements in regard to licensing and surveillance, improved track design and training environments, and increased controls over the breeding of greyhounds (7).

In practice, measuring the efforts of the industry toward improved animal welfare poses many challenges. Without existing benchmarking data, it is difficult to objectively state whether the frequency of injury events or number of fatalities over a certain period of time is acceptable or not. This study determined the reliability, alignment, and transparency of current reporting practices and thus aids in determining how committed the greyhound racing industry has been in adopting the recommendations of the independent reviews to maintain social license and improve animal welfare.

## Methods

All data and websites examined in this study were accessed between February 2021 and April 2022. The three Australian states with the highest number of active racetracks were selected for the focus of this paper. To determine this, a preliminary search was conducted via the Australian Racing Greyhound (ARG) website (8). This was compared to the venues listed on two other websites, *The Greyhound Recorder* and *The Dogs*, to ensure no racetracks were missed (9, 10). Discrepancies between the number of venues listed were found, therefore each venue was individually examined to find out which racetracks were still active as of December 2021. A venue was categorized as active if a greyhound race had occurred on its tracks within the previous 6 months or if future meetings were planned. If the last race occurred more than 6 months ago and also had no planned meetings, the venue was labeled as likely inactive. Direct evidence for official closure of venues was searched for through the venues' official websites. Where evidence could not be found via their websites, official communications on behalf of venues such as media releases or Facebook announcements on official greyhound pages were accepted as proof of permanent closure.

**Figure 1:**
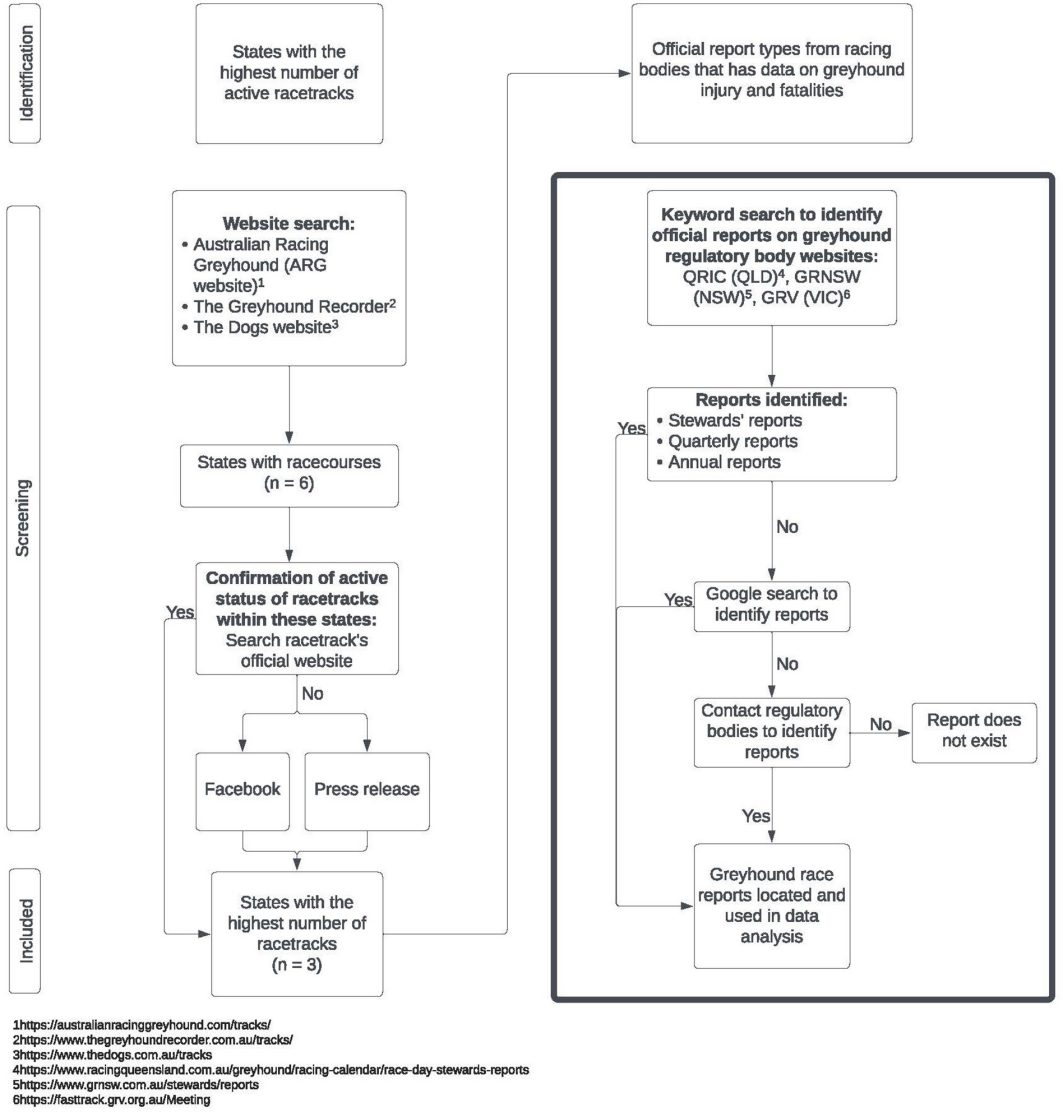
Flowchart of research process. 1 https://australianracinggreyhound.com/tracks/ 2 https://www.thegreyhoundrecorder.com.au/tracks/ 3 https://www.thedogs.com.au/tracks 4 https://www.racingqueensland.com.au/greyhound/racing-calendar/race-day-stewards-reports 5 https://www.grnsw.com.au/stewards/reports 6 https://fasttrack.grv.org.au/Meeting.

Once the three highest race volume states were selected, official reports that contained data on greyhound injuries and fatalities were identified and extracted from online archives. As part of the assessment on the transparency of published data by the industry, an inclusion criterion for the reports analyzed in this study were that the report had to be readily available to anyone with access to the internet. A preliminary search via each state's greyhound regulatory bodies' website, Queensland Racing Integrity Commission (QRIC), Greyhound Racing NSW (GRNSW), and Greyhound Racing Victoria (GRV), was conducted using the keywords “injury” and “report.” It was found that greyhound injuries and fatalities were reported in quarterly reports and annual reports in the form of summary data. If quarterly or annual reports were not able to be located via the state websites, a secondary Google search was used as a means to check if the search via official greyhound websites had returned accurate results. Keywords used were “greyhound,” “quarterly reports,” “injury reports” and “annual reports.” If the report type still could not be located, then the relative greyhound regulatory bodies were contacted to confirm the absence of reports. Another report type that matched the inclusion criteria were stewards' reports. These reports were generated whenever a race occurred and included data on individual greyhound injuries and fatalities that related to any incidents directly affecting race day. The research methodology to extract data from public websites is outlined in Figure 1.

Once the availability of the types of reports for each state was determined, reports that were dated between 2016 and 2021 were retrieved. All three types of reports were examined to determine the qualitative and quantitative data pertaining to greyhound injury and deaths. This was done by comparing changes in reporting methods within the 6-year span both intra- and interstate. Any notable changes such as partial or complete removal of the reporting of data was then descriptively analyzed for whether changes were more likely to cause a positive or negative impact in terms of animal welfare measurement and reporting.

The databases used to generate the quarterly reports were named in the reports. Attempts to gain access to these databases were made to consider several factors, such as how user friendly it was, the type of information available, and how closely recorded data resembled whole-of-life individual greyhound tracking. Where access to a database could not be obtained, efforts to find its instruction manual were done by internet search using the database name and “manual” as keywords. Either the database itself or the manual were used to descriptively analyse the aforementioned characteristics. When necessary, state racing authorities were contacted to clarify the existence and accessibility of databases.

## Results

The only report that could not be located via the outlined search methodology was GRV's quarterly reports (Table 1). GRV was contacted and confirmed the absence of quarterly reports. Annual reports published by QRIC only had a small amount of information on injuries and fatalities (Table 2). QRIC confirmed this was the correct annual report to access for the study's purpose, and more comprehensive reports were not available. Stewards' reports are generated whenever a race occurs noting individual animal events including injuries and veterinary examinations, and their data used to form the basis of summary data presented in quarterly and annual reports. Comparison between the three report types showed unique datasets that could be gained from each. Individual breakdown of greyhound injuries and death were only available via stewards' reports, whereas summary data published in quarterly and annual reports were absent from stewards' reports.

**Table 1:** Availability of report types for QLD, NSW and VIC between 2016 and 2021.

**State (regulatory body)**	**Stewards' reports**	**Quarterly reports**	**Annual reports**
QLD (QRIC)	✓	✓	✓
NSW (GRNSW)	✓	✓	✓
VIC (GRV)	✓	**X**	✓

**Table 2:** Summary of changes made to QLD, NSW and VIC stewards', quarterly, and annual reports published between 2016 and 2021.

**QLD Year**	**Stewards' reports**	**Quarterly reports**	**Annual reports**
2016–17	Injury reporting: º Scratchings º Late scratchings º Race day injuries Fatality reporting: º Deaths as part of scratchings º Race day deaths	Not published	Injury reporting: º No information on injuries Fatality reporting: º Minimal information on fatalities º Number euthanised at the GAP^1^ facilities and reason for euthanasia reported
2017–18	No significant changes	Injury reporting: °Injuries categorized by severity and reported as: °Total number of injury incidents º Total injury incidents per 1,000 starts º Injuries reported per racetrack Fatality reporting: °Chart showing percentage of injuries sustained according to the anatomical location that led to deaths º Deaths reported to the Commission by owners	Injury reporting: º No significant changes Fatality reporting: º Minimal information on fatalities º Only the number of greyhounds euthanised at GAP facilities due to being unsuitable for rehoming are reported
2018–19	No significant changes	No significant changes	No significant changes
2019–20	No significant changes	No significant changes	Injury reporting: º Information on injuries no longer available Fatality reporting: º Information on fatalities no longer available
2020–21	No significant changes	No significant changes	Injury reporting: º Information on injuries not available Fatality reporting: º Reporting limited to the number of race starts and number of euthanasia on track
**NSW**			
**Years**	**Stewards' reports**	**Injury reports** ^2^	**Annual reports**
2016/17	Injury reporting: º Scratchings º Late scratchings º Race day injuries Fatality reporting: º Deaths as part of scratchings º Race day deaths	Not published	Injury reporting: º Information on injuries not available Fatality reporting: º Reporting limited to the reason and number of euthanasia between 2016/17 while at the GAP facilities
2017/18	No significant changes	Not published	Injury reporting: º Information on injuries not available Fatality reporting: º Reporting limited to the number of GAP greyhounds euthanised between 2017/18 due to being unsuitable for rehoming º Percentage of euthanasia compared to the previous financial year
2018/19	No significant changes	Injury reporting: °Injuries categorized by severity and reported as: °Total number of injury incidents º Injury/100 raced (%) º Total injury incidents per 1,000 starts º Injury trends including comparison with previous quarter(s). Reporting of fatalities: °Fatalities reported as part of catastrophic injuries in the injury category table	No significant changes
2019/20	No significant changes	Injury reporting: °Added: °Percentage injured per category º Cumulative total per injury category º Changed “Major injuries” to “Injuries by severity”	Injury reporting: º Information on injuries not available Fatality reporting: º Information on fatalities not available
2020/21	No significant changes	Injury reporting: °Removed Minor I (0 incapacitation days), incorporating both Minor I and II together as “Minor”. In 2021: °Euthanasia when not occurring as part of a race now included °Report design/format changed for readability °Additional data on Major II and Catastrophic injuries with detailed breakdown of injuries including sex, age, distance, location on track and the racing history	Injury reporting: º Information on injuries not available Fatality reporting: º Reporting limited to the number of euthanasia on track
**VIC**			
**Years**	**Stewards' reports**	**Quarterly reports**	**Annual reports**
2016/17	Injury reporting: º Scratchings º Late scratchings º Race day injuries Fatality reporting: º Deaths as part of scratchings º Race day deaths	Not published.	Injury reporting: º Per 1000 starters Fatality reporting: º Euthanasia of VIC-owned º Race fatalities at VIC tracks º Per 1000 starters Number of euthanasia compared to the previous financial year
2017/18	No significant changes	No significant changes	No significant changes
2018/19	No significant changes	No significant changes	No significant changes
2019/20	No significant changes	No significant changes	No significant changes
2020/21	No significant changes	No significant changes	No significant changes

^1^ Greyhound Adoption Program. ^2^ Quarterly reports in NSW are known as injury reports.

The timing of quarterly reports coincides with the financial year, where the 1^st^ quarterly report (Q1) contains data generated between the 1^st^ July and the 30^th^ September of each year. Comparison of changes made to quarterly and annual reports published between 2016 and 2021 were evaluated separately for the three states, and presented in Table 2, which lists the general information available in different report types. Changes that did not alter the interpretation of information, such as the order of data presented, were not included in the results. Any changes noted are ongoing unless stated otherwise.

Regarding Queensland, although annual reports are published by QRIC, information relating to morbidity and mortality of greyhounds was minimal compared to NSW annual reports. Only the number of greyhounds euthanized whilst part of the greyhound adoption program was published. There were no data on injuries sustained in races, and none on fatalities related to racetrack injuries until the 2020–2021 report. From 2020 to 2021 the QRIC report was updated to record the number of euthanized dogs and the euthanasia rates (per 1,000 starts) for greyhounds. QRIC's annual reports were unique compared to GRV and GRNSW as the reports were not species-specific, containing information for racing greyhounds, and Standardbred and thoroughbred horses. Information pertaining to different industries were not distinctly separated and were mixed within the report.

Types and public access to the original databases was variable (Table 3). According to the Queensland Government's response to the MacSporran Report, the national database of greyhound statistics (OzChase) should be available to the public upon request and payment of a fee (5). Racing Queensland (RQ) was contacted to enquire about access and fees and indicated to the research group that the database is not for external or public use. Possible access could be granted upon an approved Right To Information (RTI) application. In NSW, data is recorded in the Greyhound Examination Database (GED) by the Commission's On-Track Veterinarians (OTVs). The number of race starts and race meetings are recorded in OzChase and OneGov. These databases are not open for external or public use. In contrast, VIC's FastTrack database allows access for both registered and unregistered database users, however, both user types have access to the same level of information.

**Table 3:** Databases used for greyhound tracking.

**State**	**Database**	**Tracking period**	**Access**	**Developed/maintained by**
QLD	1. OzChase	From birth to leaving the racing industry	Available to the public upon request and payment of a fee (5). However, requires an approved RTI application	OzChase was developed by GRNSW in a joint venture arrangement with Racing and Wagering Western Australia (10), who also designed, built, hosts and maintains the system (10)
NSW	1. Greyhound Examination Database (GED), 2. OzChase 3. OneGov	From birth to leaving the racing industry	1. On-track vets can access GED. 2. OzChase (as above) 3. OneGov is part of Greyhound Welfare and Integrity Commission's (GWIC) business systems and is available to Commission's staff	1. GED is managed by Faculty of Engineering and Information Technology at the University of Technology Sydney (UTS) (11) 2. As above 3. OneGov is managed by GWIC
VIC	GRV FastTrack	From birth to leaving the racing industry	Access available online. Registration does not grant a different level of access compared to non-registered users	GRV

## Discussion

Racing greyhounds are subjected to increased forces on their body that may result in injury, with a subsequent risk of debilitation or catastrophic consequences such as death (12, 13). In order to determine the true reality of injuries greyhounds sustain during their racing careers, independent analysis of morbidity and mortality data remains an animal welfare priority. In this study, the reporting methods utilized in QLD, NSW and VIC by the greyhound industry were evaluated for accessibility, consistency, and reliability. It was found that the three major forms of reports containing relevant greyhound mortality and morbidity data were stewards' reports, quarterly reports, and annual reports. Access to these reports were relatively easy as they could be found either through internet searches using the keywords outlined in the methodology, or by using the search function via the respective state greyhound regulatory body's website. Evaluating consistency and transparency of reporting aids in gauging how focused the industry has been on its obligation to animal welfare measures, and whether positive progress was occurring. Previously, a 2016 UK study found the lack of transparency in the greyhound industry was associated with reduced confidence that injury data is being used to improve greyhound welfare (14).

A common statement featured within NSW annual reports was the promise to heavily invest in welfare activities and to have detailed injury reporting requirements (15). However, injury and fatality data in annual reports became brief, if not almost non-existent from 2018 onwards (Table 2). Therefore, the NSW annual reports were of limited use when assessing morbidity and mortality data, which is discordant with a stated aim of focusing on animal welfare when preparing these reports. Another outcome of this research was to highlight that without a national standard for greyhound reporting there is a substantial interstate variation in report quality and content. Analysis of the variation between different state reports demonstrated what reporting strategies should be recommended as a requirement. QLD annual reports had no information relating to injuries and ranged from having brief to no information on euthanized dogs. Furthermore, data on different species were presented within the same report, which were divided by topic and not by species. It would be preferable to have species-specific reports like NSW and VIC or have separate sections for different industries to ensure relevant data is not obscured by mixed information. VIC annual reports fared the best in terms of content, reporting both the total number of injuries and fatalities, and also the standardized number per 1,000 starters. However, GRV does not publish quarterly reports, whereas QLD and NSW do. The QLD quarterly reports remained the most consistent across the researched time period, closely followed by NSW's injury reports. Quarterly reports provide highly relevant information such as the total number of injuries and fatalities, standardized data (incidents/100 raced and incidents/1,000 starts), trend comparisons with other quarters, and analyses where applicable. Commendably, NSW was the only state to publish dog euthanasia that resulted from reasons other than a racing-related incident. Our recommendation is that other states should be encouraged to follow suit, as without this data, greyhounds that die or are euthanized off-track after sustaining injury at race meetings or due to trials are missed.

Ideally, access to raw data to independently evaluate trends without industry input would allow the most transparency, reducing the possibility of bias in the assessment of injury and fatality rates. Having this information would also allow replication of industry processes when generating official reports to evaluate the accuracy of published data. Access to the national OzChase database was requested through QRIC who subsequently indicated to the research team that applications for access must be through Racing Queensland (RQ). Despite statements by the Queensland Government that they will adhere to recommendations for the database to be made available to the public upon request and a fee, the response from RQ when contacted was that OzChase was not available for external or public use. RQ did, however, point to possible access through an approved RTI application, under Freedom of Information legislation, but this process was outside the scope of this study's methodology.

Similarly, the three databases used by GRNSW are also restricted, meaning that independent analysis can only rely on information made public by the industry. In comparison, public access is available from Victoria's GRV's FastTrack dataset. User registration is also possible and is instantaneous but does not offer more detailed information in terms of greyhound morbidity and mortality data compared to accessing the site as an unregistered user. It does, however, grant user access to additional services such as applying to participate as an owner, catcher, attendant, owner-trainer or public trainer, as well as viewing Greyhound Adoption Program (GAP) dogs available for adoption.

All databases only track greyhounds from birth until they leave the racing industry, essentially relieving any accountability of the industry for reporting once a dog is registered as having left the sport. Therefore, dogs that are euthanized after retiring from the racetrack due to injuries or lack of rehoming are potentially not recorded. This suggests that the number of greyhound morbidity and mortality events are potentially higher than what is captured in current datasets. This suggests whole-of-life tracking for racing greyhounds could be mandated to ensure industry data reflect the true impact of greyhound racing on dog welfare.

Without access to the raw data within a database, an alternate method to determine dog status is by collating and analyzing injury and mortality data, including reviewing what is produced in stewards' reports for individual race meetings and tracks. QRIC stewards do not consistently follow the same format when generating stewards' reports, which results in obscure information in some of the reports. For example, information may be categorized differently and sometimes incorrectly, such as having duplicate information entered in both scratchings and late scratchings. Another notable example is the use of the title “animal actions” for the collective reporting of injuries, deaths, and trials instead of separate categories, which makes data retrieval difficult. In NSW and VIC, stewards' reports follow consistent formatting. A recommendation of this research would be to standardize and harmonize steward report format and terminology between states, specifically harmonization of QLD steward's reports with other states. However, analysis of steward's report data still requires significant effort due to the vast number of races that have occurred and the need to take care when extracting information of interest. These problems could be alleviated by having access to the databases that the racing report information is generated from, or by creating a nationally harmonized database which is publicly accessible.

Several recommendations have been identified through this study which would improve greyhound industry data reporting transparency and accessibility. Reports should follow consistent formatting, especially within the same state. Depending on the discretion of state greyhound regulatory bodies, the availability of information greatly differs. These problems could be solved by using uniform templates across all states, which would enforce consistency and reporting of essential data. VIC should publish quarterly reports, as these provide significantly more detail in regard to morbidity and mortality data compared to annual reports. Another improvement would be for all states to follow NSW in publishing euthanasia that is carried out not as part of a race, especially since there is no whole-of-life tracking for greyhounds. Similarly, tracking from birth to death of racing dogs is greatly encouraged to ensure owners and trainers, as well as the industry at large, remain accountable for the whole lifespan of the animal. However, it is worth noting that this presents logistical challenges, as the authority of racing regulatory bodies is restricted to dogs within the racing system. Thus, when greyhounds are retired and the ownership changes, authorities lose legislative jurisdiction over them. As it is up to the new owners to report outcomes, and they may not be inclined to do so, accurate statistics for post-racing outcomes may be challenging to acquire. For animals successfully rehomed as pets, further tracking is likely not informative for animal welfare, but there is a risk that classifying animals as retired could be used to mask euthanasia or poor welfare outcomes. In comparison, the horse racing industry have been making progress toward better equine traceability through requiring 6 monthly updates on all active Thoroughbred horses and annual updates for those registered with the breeding community (16). Annual reports for Thoroughbred racing show the percentage of horses that have retired from racing and those that have reached its end of life (16). It is highly recommended for the greyhound industry to follow suit.

A recent development in NSW is the announcement of an e-tracking system that when implemented will be aimed at monitoring the location and welfare of greyhounds, which is a promising step toward greater traceability of animals (17). Echoing the recommendations put forth by the NSW Government's Greyhound Industry Reform Panel, priority should be given to minimize and ultimately achieve zero unnecessary euthanasia of greyhounds (7). The panel's suggestions to implement enforceable regulations, new offenses, and stronger penalties to deter animal cruelty complements the aim to decrease injury and death of animals (7). The fact that the information the public is privy to is controlled by the industry, and raw data is not publicly accessible, casts doubt on the reliability of the information retrieved. Hence, access to databases where pertinent information related to assessing animal welfare measures within the industry should be provided to the general public or research groups as a matter of transparency and public good. If privacy issues via database access is a concern for the industry, having tiered access based on approval level could be used. For both public and research purposes, limiting access to sensitive information not required for the intended purpose may be another option.

A number of limitations exist for this study. Only data from three states were retrieved for analysis, however these were the most active dog racing states in Australia. Due to the inability to access raw data from the databases, published reports which were reviewed were essentially self-reported data which cannot be independently verified. Therefore, information that is assessed may be subject to biases such as reporting bias, where data and analyses favorable to the industry are selectively published. Although biases and errors such as mistakes in data entry exist for any data collection process, the reliability of the published reports could not be independently evaluated in this study, as information on the methodology used in report generation was not available, including how the data were gathered, archived, analyzed, interpreted, and reported. Apart from NSW, no other states publish euthanasia not as part of a race (18). This means that unnecessary euthanasia of healthy greyhounds is not routinely accounted for and unable to be reliably traced. This is another limitation that results from the absence of whole-of-life tracking. Only reports between 2016 and 2021 were examined. Therefore, injury and fatality trends before 2016, and any advancements in animal welfare instigated after 2021 were not included. Furthermore, QLD and NSW only started publishing quarterly reports in 2018, and VIC do not publish these reports at all, which limited the data available for comparison. Combined with the inability to access raw data, the alterations and gaps in report style and format made it difficult to formulate benchmarking data for greyhound injuries and fatalities. A limitation identified only in QLD annual reports was that data were separated by topic and not by species, whereas NSW and VIC had species specific reports, which affects data integrity.

## Conclusion

This study identified the types and content of greyhound racing reports in the three largest dog racing states within Australia. Although significant improvements in some reporting metrices and styles were noted since 2015, there remains considerable opportunities to improve transparency, reliability, harmonization, and standardization of greyhound data relevant to animal welfare assessment. Our study identified several opportunities to improve data reporting practices across states, notably the harmonization of the type, style, and content of stewards, quarterly, and annual dog racing reports. Further, whole-of-life tracking, and reporting of racing dog euthanasia not related to racing events would be a significant animal welfare metric of benefit to industry accountability and social license. A gold standard aim would be to make raw data, which is fed into industry reports, as well as the methodology for analysis publicly available. A nationally controlled database would also be a significant asset in allowing standardization, and independent monitoring of the dog racing industry within Australia.

## Data availability statement

The original contributions presented in the study are included in the article/supplementary material, further inquiries can be directed to the corresponding author.

## Author contributions

VC conducted the data extraction analysis and drafted the manuscript. JH assisted with the data analysis and figure construction. KD and RA drafted and reviewed the manuscript. VC, JH, KD, and RA helped with the study design. All authors contributed to the article and approved the submitted version.

## Conflict of interest

The authors declare that the research was conducted in the absence of any commercial or financial relationships that could be construed as a potential conflict of interest.

## Publisher's note

All claims expressed in this article are solely those of the authors and do not necessarily represent those of their affiliated organizations, or those of the publisher, the editors and the reviewers. Any product that may be evaluated in this article, or claim that may be made by its manufacturer, is not guaranteed or endorsed by the publisher.
